# Altered Osteocyte-Specific Protein Expression in Bone after Childhood Solid Organ Transplantation

**DOI:** 10.1371/journal.pone.0138156

**Published:** 2015-09-21

**Authors:** Renata C. Pereira, Helena Valta, Navdeep Tumber, Isidro B. Salusky, Hannu Jalanko, Outi Mäkitie, Katherine Wesseling Perry

**Affiliations:** 1 Department of Pediatrics, David Geffen School of Medicine at UCLA, Los Angeles, United States of America; 2 Children's Hospital, Helsinki University Central Hospital, University of Helsinki, Helsinki, Finland; 3 Folkhälsan Research Center, Helsinki, Finland; 4 Department of Molecular Medicine and Surgery and Center for Molecular Medicine, Karolinska Institutet, Stockholm, Sweden; Universidade de Sao Paulo, BRAZIL

## Abstract

**Background:**

Bone fragility is common post solid organ transplantation but little is known about bone pathology on a tissue level. Abnormal osteocytic protein expression has been linked to compromised bone health in chronic kidney disease (CKD) and immunosuppressant medications may impact osteocyte function.

**Methods:**

Transiliac bone biopsies were obtained from 22 pediatric solid organ allograft recipients (average age 15.6 years) an average of 6.3 ± 1.2 years after transplantation and from 12 pediatric pre-dialysis CKD patients (average age 13.2 years). Histomorphometry and immunohistochemistry for FGF23, DMP1, sclerostin, and osteopontin were performed on all biopsies.

**Results:**

FGF23 and sclerostin were increased in transplant recipients relative to non-transplant CKD, regardless of the type of allograft received and despite, in the case of liver and heart recipients, a higher GFR. Bone DMP1 expression was higher in liver or heart than in kidney recipients, concomitant with higher serum phosphate values. Osteopontin expression was higher in CKD than in transplant recipients (p<0.01). Bone FGF23 and sclerostin correlated directly (r = 0.38, p<0.05); bone FGF23 expression and osteoid thickness correlated inversely (r = - 0.46, p<0.01).

**Conclusions:**

Solid-organ transplantation is associated with increased FGF23 and sclerostin expression. The contribution of these findings to compromised bone health post transplantation warrants further evaluation.

## Introduction

Skeletal problems are common in pediatric recipients of solid organ allografts, with fractures in general occurring 6 times as commonly as in the general population and vertebral fractures, specifically, occurring at even higher rates [1,2]. While the deleterious effects of immunosuppressive agents contribute to skeletal morbidity in all allograft recipients, the residual effects of renal osteodystrophy, which is associated with bone fragility and skeletal deformities in children with chronic kidney disease (CKD) [3,4], may also contribute to skeletal morbidity post-renal transplantation.

Osteocytes are key regulators of bone modeling and remodeling [5] and current data suggest that CKD is associated with abnormal expression of different osteocytic proteins [6,7], some of which, such as fibroblast growth factor 23 (FGF23) and dentin matrix protein 1 (DMP1), play a role in regulating skeletal mineralization and others of which (namely sclerostin) regulate osteoblast differentiation [5,8]. Abnormalities in osteocytic protein expression occur in early CKD, before abnormalities in mineral ion, vitamin D, and parathyroid hormone (PTH) concentrations are apparent and coincide with early changes in bone turnover and mineralization [6,7]. Some data also suggest that circulating FGF23 levels are affected by immunosuppressant medications [9]; however, the effects of immunosuppressant agents and their interaction with decreased renal function on osteocytic protein expression have not been evaluated. Thus, in order to gain more knowledge on the pathological processes contributing to skeletal fragility after pediatric solid organ transplantation, in which the presence of CKD and the use of immunosuppressant agents may both contribute to skeletal pathology, we evaluated osteocytic protein expression and bone histology in a cohort of kidney, liver, and heart transplant recipients and in subjects with pre-dialysis CKD.

## Methods

### Patients

This cross-sectional analysis represents data from two institutions. Transiliac bone biopsies from pediatric kidney, liver, and heart transplant recipients were obtained from 22 children (mean age: 15.6 years; age range: 7.6 to 19.8 years) with a history of kidney (n = 8), liver (n = 9), or heart (n = 5) transplantation as a part of clinical evaluation for suspected osteoporosis at the Children’s Hospital, Helsinki University Central Hospital, where all pediatric solid organ transplantations and post-operative care are centralized in Finland. Study participants were, on average, 9.4 ± 1.2 years of age at the time of transplantation and bone biopsies were performed 6.3 ± 1.2 years post transplantation. The bone histomorphometric, biochemical, and bone density data from a subset of 19 of these individuals have been previously reported [10]; the current study presents these individuals as well as 3 additional (2 kidney and 1 heart) transplant recipients who subsequently underwent bone biopsy. Bone biopsies from 12 pre-dialysis CKD patients (mean age: 13.2 years; age range: 2.2 to 19.8 years) were obtained at UCLA as part of a previously reported study characterizing the spectrum of renal osteodystrophy in pediatric pre-dialysis CKD [11]. This study was approved by the both the UCLA and the University of Helsinki institutional review boards. Due to the use of de-identified historical samples, informed consent was waived by both institutions.

All solid organ transplant recipients had received glucocorticoids since transplantation and were receiving low-dose alternate-day oral glucocorticoids (methylprednisolone). Total cumulative (mg) and weight-adjusted (mg/kg) glucocorticoid doses as well as glucocorticoid exposure (mg/kg/days) during the previous 3 years were calculated [10]. None of the children had been treated with aluminum-containing phosphate binders or bisphosphonates. None of the patients had received growth hormone treatment.

### Bone Biopsy and Histomorphometry

Full thickness bone biopsies were obtained from the anterior iliac crest using a modified Bordier trephine (0.5 cm diameter) needle after double tetracycline-labeling. Specimens were fixed in 70% ethanol, dehydrated in alcohol, cleared with xylene, and embedded in methylmethacrylate. Static histomorphometric parameters were evaluated in undecalcified 5 μm sections stained with Toluidine blue; tetracycline labeling was assessed in unstained 10 μm sections.

Primary bone histomorphometric parameters were assessed in trabecular bone under 20x magnification using the OsteoMetrics system (OsteoMetrics, Decatur, GA) and classified under the Turnover, Mineralization and Volume (TMV) system [12]. Normal values for histomorphometry were previously obtained from double-tetracycline labeled iliac crest specimens from 31 pediatric patients with normal kidney function undergoing elective orthopedic surgery [13].

### Immunohistochemistry and Quantification of Bone FGF23, DMP1, Sclerostin, and Osteopontin Expression

The technique for immunohistochemical detection of protein in bone was adapted from a previously reported method [14]. In brief, 5 μm sections of bone tissue were de-plastified in xylene and chloroform, rehydrated in graded alcohol solutions, and partially decalcified in 1% acetic acid. Endogenous peroxidase activity was quenched in 3% hydrogen peroxide/methanol solution. Non-specific binding was blocked in avidin-biotin solution and in 5% normal horse serum with 1% bovine serum albumin. Sections were incubated with affinity purified polyclonal goat anti-human FGF23(225–244) (Immutopics Intl, San Clemente, California) (dilution 1:500), monoclonal anti-human DMP1 (LFMb31)(62–513) (Santa Cruz Biotechnology, Inc., Santa Cruz, CA) (dilution 1:50), monoclonal anti-human sclerostin (R&D Systems, Minneapolis, MN) (dilution 1:500), or monoclonal osteopontin (LFMb-14) (Santa Cruz Biotechnology, Inc., Santa Cruz, CA) (dilution 1:500) primary antibody overnight at 4°C in a humidified chamber. Sections were then incubated with biotinylated anti-goat (Vector, Burlingame, CA, USA), anti-mouse (Sigma-Aldrich, St. Louis, MO), or anti-rabbit (Sigma-Aldrich, St. Louis, MO) antibody; incubated for 30 minutes with StreptABC Complex/HRP kit (Vector) follow by AEC substract Chromogen (Dako, Carpinteria, CA); and counterstained with Mayer hematoxilin (Sigma-Aldrich).

For immunohistochemical analysis, iliac crest bone biopsy specimens from 5 adolescent and young adult subjects with normal renal function were used to represent a population of healthy controls [6]. Negative controls were performed for each bone section by omitting the primary antibody. Reproducibility was insured by repeating the immunohistochemistical analysis on all specimens. FGF23, DMP1, and osteopontin were assessed in the entire area of trabecular bone; however, since sclerostin was found to be expressed almost exclusively in the cortex, its expression was assessed only in cortical bone. Immunoreactivity for FGF23 was quantified by counting the number of osteocytes expressing FGF23 in one 5 μm section of trabecular bone and normalizing this number by tissue area. Immunoreactivity for DMP1, osteopontin, and sclerostin was quantified using the Ariol scanning system and also normalized by total (for DMP1 and osteopontin) tissue or cortical (for sclerostin) tissue area. All slides were scanned at 20x magnification with a red filter and digitized (Applied Imaging Inc., San Jose, CA). Analyzed fields were manually selected to avoid areas with tissue damage occurring during immunostaining. Staining was expressed as pixels/mm^2^ [15–18]. All analyses were performed with the MultiStain script.

### Biochemistry

Blood and urine samples were collected at the time of the bone biopsy. Plasma concentrations of ionized calcium, phosphate, alkaline phosphatase, and creatinine were determined using standard methods. In the transplant recipients, glomerular filtration rate (GFR) was measured by 51-labeled chromium ethylenediaminetetraacetic acid clearance. In the pre-dialysis CKD population, GFR was estimated using the modified Schwartz equation [19]. In the transplant recipients, serum concentrations of 25(OH)vitamin D were determined by high performance liquid chromatography followed by UV detection (HP 1100 Liquid Chromatograph, Agilent Tech, Santa Clara, CA, USA). Radioimmunoassay was used to determine 25(OH)vitamin D values in non-transplant CKD patients [20].

### Statistical Analysis

Measurements for normally distributed variables are reported as mean ± standard error (SE); median values and interquartile range are used to describe non-normally distributed variables. Ranges are presented where noted in the text. The Wilcoxon signed rank test was used to assess inter-group differences. Relationships between biochemical, bone histomorphometric, and immunohistochemical parameters were assessed by Spearman correlation coefficients. All statistical analyses were performed using SAS software (SAS Institute Inc., Cary, NC) and all tests were two-sided. A probability of type I error less than 5% was considered statistically significant and ordinary p values are reported.

## Results

### Patients and Biochemical Values

Twenty-two pediatric solid organ recipients, all of whom were treated with triple immunosuppressant therapy consisting of a calcineurin inhibitor, methylprednisolone, and an antimetabolite, and 12 pediatric pre-dialysis CKD patients were enrolled in the current study. All transplant recipients received between 400 and 800 IU of vitamin D_3_ daily; no subjects received calcitriol or other active vitamin D sterols. As has been previously described, all children had received glucocorticoids since transplantation and were receiving low dose alternate-day oral glucocorticoids (methylprednisolone) at the time of biopsy. Cumulative weight-adjusted glucocorticoid doses as well as glucocorticoid exposure during the previous 3 years did not differ between patients with liver or heart and patients with kidney allografts. Other demographic and biochemical data are displayed in Table 1. In general, patients were within the adolescent age range and age did not differ between groups. GFR and PTH values did not differ between kidney transplant recipients and the cohort with pre-dialysis CKD. Serum calcium concentrations were higher while serum phosphorus and alkaline phosphatase values were lower in the kidney transplant recipients.

**Table 1:** Demographic and biochemical parameters in transplant and CKD patients.

	Pre-dialysis, non-transplant CKD (n = 12)	Kidney transplant (n = 8)	Liver or Heart Transplant (n = 14)	Normal range
Age (y)	13.2 ± 1.4	16.9 ± 2.0	14.8 ± 0.8	
Gender	8M/3F	4M/4F	8M/6F	
Time since transplantation (y)	NA	8.9 ± 2.2	4.8 ± 1.2	
GFR (ml/min/1.73m^2^)	54.7 ± 5.9	42.5 ± 3.8	72.8 ± 4.4 *	
Cumulative weight-adjusted glucocorticoid dose (mg/kg)	NA	53.9 (43.9, 128.4)	82.9(49.9, 110.3)	
Weight-adjusted glucocorticoid exposure (mg/kg/d)	NA	0.05 (0.04, 0.13)	0.12 (0.05, 0.19)	
Calcium (mg/dl) ***	9.4 ± 0.1	10.3 + 0.1 **	10.0 ± 0.1	8.4–10.2
Phosphorus (mg/dl)	4.3 ± 0.3	3.5 ± 0.3 **	4.0 ± 0.1	2.2–4.7
Alkaline Phosphatase (IU/l)	229 (138, 343)	108 (88, 146) **	138 (86, 216)	31–103
25(OH)vitamin D (pg/ml)	27 ± 2	31 ± 10	30 ± 3	>30
PTH (pg/ml)	68 (39, 155)	73 (54, 79)	33 (29, 50) *	8–73

* Indicates a difference between kidney transplant recipients and liver or heart transplant recipients (p<0.05)

**Indicates a difference between kidney transplant recipients and pre-dialysis CKD patients (p<0.05)

***Values of transplant recipients converted from mmol/l to mg/dl

As has been previously reported (Tamminen et al, Pediatric Nephrology 2014), pediatric transplant patients underwent bone biopsy based on a suspicion of secondary osteoporosis was based on low areal bone mineral density measured by DXA and/or fracture history. Vertebral fractures were diagnosed by plain spinal radiographs and loss of vertebral height ≥20% was used for diagnosis. In the current cohort, which consisted of 22 allograft recipients, 12 (55%) (7 liver, 3 heart, and 2 kidney transplant recipients) had low (<−2.0) lumbar spine BMD Z-score. Low total hip BMD Z-score was observed in 7 (31%), including 3 kidney and 4 liver allograft recipients. Four (18%) of the children, all liver allograft recipients, had sustained peripheral fractures (in humerus, radius, fibula, and metatarsus; 1 in each), whereas vertebral compressions were found in more than half (59%) of all allograft recipients.

To assess the interaction between GFR and transplantation on osteocytic protein expression, patients with renal allografts were compared to those who were status post other solid organ transplantation. Overall, GFR was lower and PTH levels were higher in kidney transplant recipients than in recipients of other types of solid organs. However, other biochemical values did not differ between these two groups of patients.

### Bone Histomorphometry

In all three groups of patients, bone volume was within the normal range. Median values for bone formation rate were also within the normal range; however, values tended to cluster within the lower end of the normal range. Osteoid accumulation was also in the normal range, although values for this parameter tended to be in the upper limits of normal. Mineralization rates, as measured by osteoid maturation time (OMT), were also in the upper range of normal. With the exception of mineralization lag time, which was slightly higher in kidney transplant recipients than in the recipients of other solid organ transplants, bone histomorphometric indices were similar between kidney transplant recipients, recipients of other solid organs, and non-transplant CKD patients (Table 2).

**Table 2:** Bone histomorphometric parameters in transplant and CKD patients.

	Pre-dialysis, non-transplant CKD (n = 12)	Kidney transplant (n = 8)	Liver or Heart Transplant (n = 14)	Normal range [13]
Volume				
Bone volume (BV/TV) (%)	29.3 ± 2.6	24.8 ± 1.8	21.7 ± 2.2	8.9–34.4
Trabecular thickness (Tb.Th) (um)	132 ± 11	110 ± 8	113 ± 7	91–175
Trabecular separation (um)	324 ± 20	342 ± 31	441 ± 37*	351–737
Mineralization				
Osteoid volume (OV/BV) (%)	7.2 ± 3.4	2.2 ± 0.5	2.0 ± 0.3	0.2–5.8
Osteoid surface (OS/BS) (%)	24.8 ± 5.4	16.4 ± 3.4	15.8 ± 2.7	4.3–31.7
Osteoid thickness (O.Th) (um)	11.4 ± 2.5	6.9 ± 0.6	7.0 ± 0.3	2.0–13.2
Osteoid maturation time (OMT) (d)	9 (8, 19)	15 (16, 18)	11 (10, 13) **	1.2–11.5
Mineralization lag time (MLT) (d)	25 (13, 44)	38 (33, 63)	28 (22, 34)	2.3–63.8
Turnover				
Bone formation rate (BFR/BS) (um^3^/um^2^/y)	14.7 (8.7, 28.5)	9.8 (2.8, 18.4)	10.1 (3.5, 26.4)	8.0–73.4
Eroded surface (ES/BS) (%)	6.9 ± 2.0	2.0 ± 0.4	3.1 ± 0.8	0.5–4.3

* Indicates a difference between pre-dialysis CKD patients and liver or heart transplant recipients (p<0.05)

** Indicates a difference between kidney transplant recipients and liver or heart transplant recipients (p<0.05)

### Bone Protein Expression

**Fig 1:**
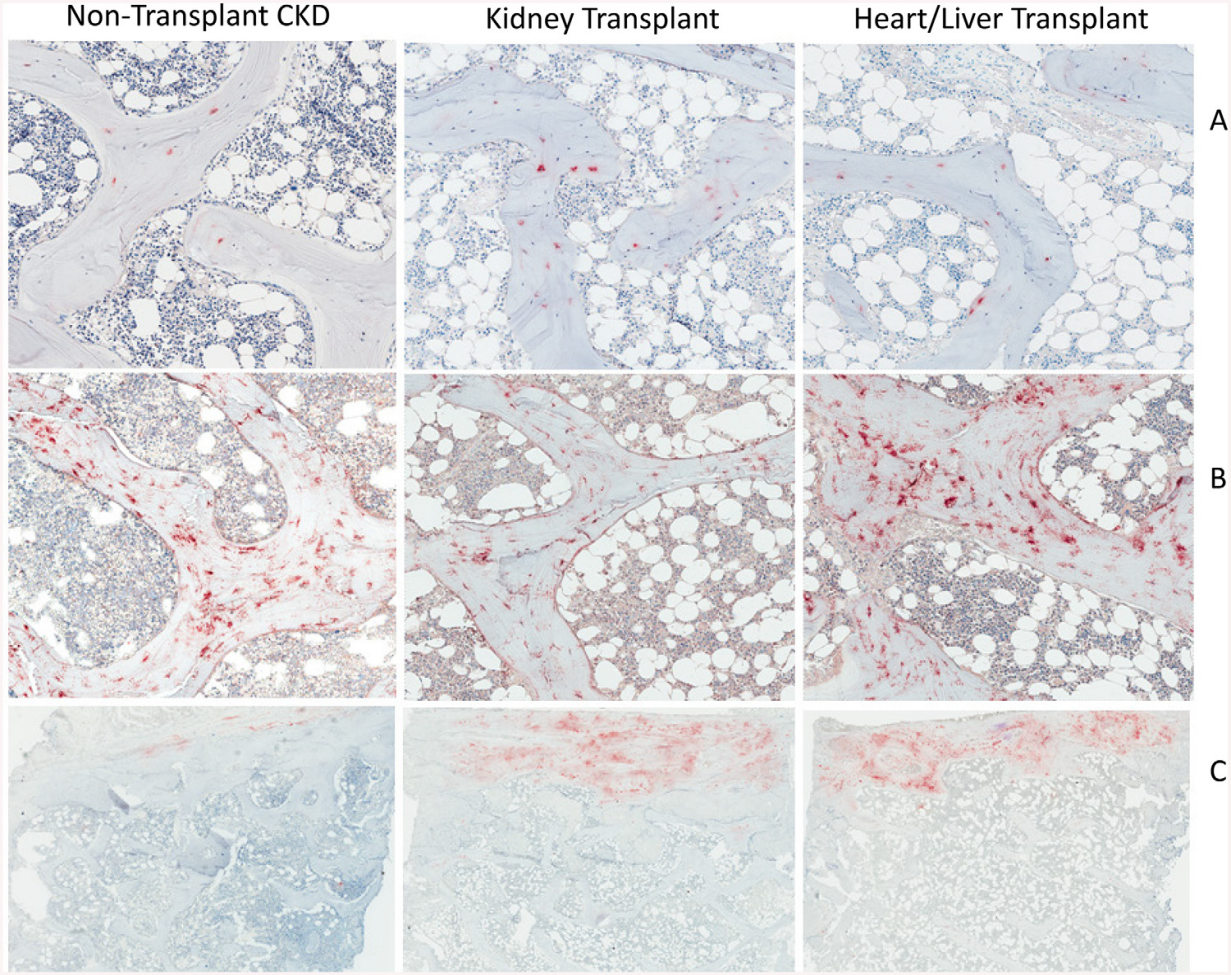
Bone (A) FGF23; (B) DMP1 and (C) sclerostin expression in representative samples from a non-transplant CKD patient, a kidney transplant recipient, and a heart transplant recipient.

**Fig 2:**
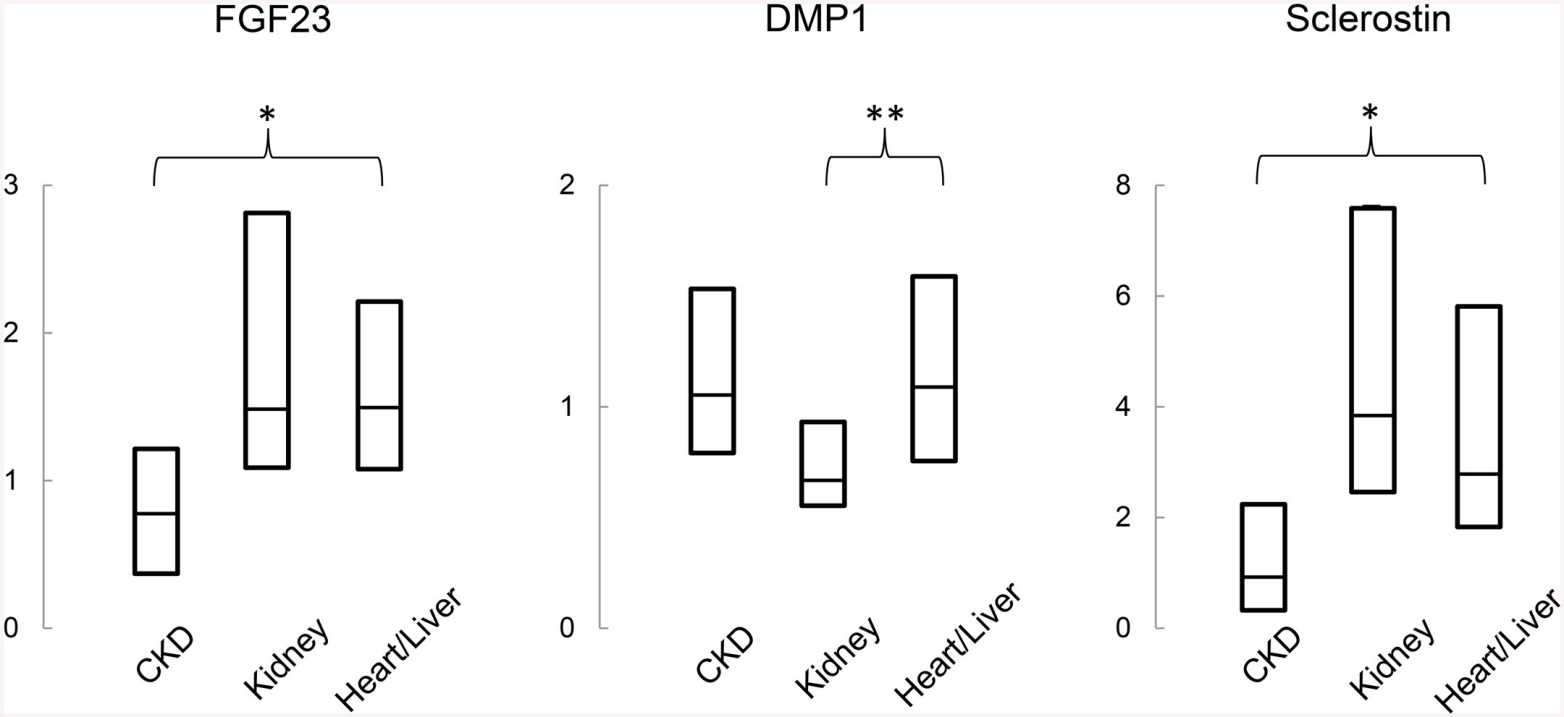
Quantification of bone (A) FGF23; (B) DMP1 and (C) sclerostin expression in non-transplant CKD patients, kidney transplant recipients, and liver or heart transplant recipients. The asterisk represents a difference between allograft recipients and non-transplant CKD patients; the double asterisk represents a difference between kidney and heart or liver allograft recipients.

Bone FGF23 was expressed in cell bodies in discrete clusters of osteocytes at the trabecular edges and expression of the protein was higher in pre-dialysis non-transplant CKD patients than in healthy controls (p<0.05 between groups) [6]. Bone FGF23 expression was further increased in recipients of solid organ transplantation as a whole (p<0.05 from pre-dialysis CKD patients). Interestingly, although GFR was lower in kidney transplant recipients than in the recipients of liver and heart allografts, no difference in bone FGF23 expression were detected between these two groups (Table 3, Figs 1 and 2).

**Table 3:** Protein expression, as determined by immunohistochemistry, in pediatric transplant and CKD patients.

	Pre-dialysis, non-transplant CKD (n = 12)	Kidney transplant (n = 8)	Liver or Heart Transplant (n = 14)	Normal range [6]
Bone FGF23/tissue area (osteocytes/mm^2^)	0.8 (0.4, 1.2)	1.5 (1.1, 2.8) **	1.5 (1.1, 2.2)**	0.3 ± 0.1
Bone DMP1/T.Ar (pixels/mm^2^)	1.06 (0.81, 1.52)	0.68 (0.57, 0.92)	1.09 (0.77, 1.58) *	0.68 ± 0.18
Bone sclerostin/cortical area (pixels/mm^2^)	0.9x10^-4^ (0.4x10^-4^, 2.2x10^-4^)	3.9x10^-4^ (2.5x10^-4^, 7.6x10^-4^)**	2.8x10^-4^ (1.9x10^-4^, 5.8x10^-4^)**	6.7x10^-4^ (2.2x10^-4^, 34.5x10^-4^)
Bone osteopontin (pixels/mm^2^)	0.052 (0.034, 0.070)	0.038 (0.028, 0.052)**	0.031 (0.024, 0.037)**	0.040 ± 0.009

* Indicates a difference between kidney transplant recipients and liver or heart transplant recipients (p<0.05)

**Indicates a difference between transplant recipients as a group (liver, heart, or kidney) and pre-dialysis CKD patients

In contrast to FGF23, bone sclerostin expression in controls and in all patients, when present, was primarily in cortical bone. Although cortical sclerostin expression correlated with bone FGF23 expression in all patients (r = 0.38, p<0.05), in contrast to bone FGF23, cortical sclerostin tended to be lower in non-transplanted CKD patients than in controls (p = 0.056). However, similar to bone FGF23, cortical sclerostin expression was increased in transplant recipients as compared to non-transplanted CKD patients (p<0.05). Bone sclerostin expression did not differ between kidney and liver/heart allograft recipients. Osteopontin was primarily expressed in the matrix of mineralized bone, in lines following cement lines, rather than in osteocytes themselves. Osteopontin expression was higher in CKD than in transplant recipients (p<0.01); however, similar to cortical sclerostin expression, osteopontin expression did not differ between kidney transplant and liver/heart transplant recipients.

DMP1 immunoreactivity was present in osteocyte cell bodies and in dendritic processes throughout bone. In contrast to FGF23, sclerostin, and osteopontin expression, DMP1 immunoreactivity in transplant recipients differed based on organ type received; kidney transplant recipients had lower DMP1 expression than did their counterparts who had received liver or heart allografts (p<0.05 between groups). DMP1 expression in liver and heart transplant recipients were similar to values observed in non-transplanted pre-dialysis CKD patients.

Bone FGF23 expression correlated with parameters of skeletal mineralization; osteoid thickness inversely correlated with bone FGF23 expression (r = - 0.46, p<0.01) when all subjects (kidney transplant recipients, liver or heart transplant recipients, and non-transplant CKD patients) were considered together; similar relationships between osteoid thickness and bone FGF23 expression were observed when each subgroup was considered individually. No relationship between bone formation rate and FGF23, DMP1, or sclerostin was observed. No differences in expression of FGF23, DMP1, sclerostin, or osteopontin were observed between transplant patients with clinical fractures (n = 13) and those without (n = 9).

## Discussion

The current study demonstrates that therapy with immunosuppressive agents is associated with an increase in bone FGF23 and sclerostin immunoreactivity, regardless of the type of allograft received and despite, in the case of liver and heart allograft recipients, higher GFR. Bone DMP1 expression, by contrast, appears to be affected by type of solid organ received, with higher than normal values in liver and heart allograft recipients than in kidney transplant recipients. Osteopontin expression appears to be lower in transplant recipients than in CKD patients. An inverse correlation between bone FGF23 expression and osteoid accumulation (osteoid thickness) was observed in this cohort of patients, similar to the previously reported relationship between bone FGF23 and histomorphometric parameters of mineralization in pre-dialysis CKD and dialysis patients [6,7].

While the implications of these findings for bone biology and systemic complications remain to be evaluated, the current study highlights the importance of transplant status on bone FGF23 expression. While bone FGF23 has been previously shown to be significantly increased in pre-transplant CKD patients, this is the first study to examine bone FGF23 expression in transplant recipients. The overall reason for altered osteocytic protein expression in CKD in general, and increased osteocytic FGF23 expression, in particular, remains unknown since these changes occur prior to the development of any detectable abnormalities in circulating mineral ion or hormone concentrations [21]; however, changes in osteocyte protein expression suggest that damage occurs to bone that is not detectable by traditional markers of mineral metabolism or by histomorphometry. In this study, bone from all solid organ allograft recipients had higher FGF23 expression than did bone from CKD patients. The reason behind this relationship cannot be fully ascertained from a cross-sectional study; however, glucocorticoid therapy has been related to increased circulating FGF23 values in cross-sectional studies [9] and direct stimulation of osteocytic FGF23 expression by glucocorticoids may partly explain these increased values. The transplant recipients in the current study, however, also received treatment with calcineurin inhibitors and antimetabolites which the pre-dialysis CKD patients did not receive. How these agents may affect osteocytic FGF23 expression is unknown.

Although immune suppression itself, as noted in the increased osteocytic bone expression apparent in liver and heart recipients in the current study, appears to increase bone FGF23 expression, decreased GFR does not appear to compound the increased expression associated with immune suppression. However, although liver transplant and heart transplant recipients appeared to be the optimal control group to differentiate the relative effects of CKD and immune suppression on bone FGF23 expression in renal transplant recipients, it is important to note that these patients also had decreased renal function, with an average GFR of 73 ml/min/1.73m^2^. This level of decreased kidney function (CKD stage 2) has been associated with increases in both circulating FGF23 levels [21] and increased bone FGF23 expression [6]. Since even mild CKD has been associated with marked increases in bone FGF23 expression, the increased bone FGF23 expression in the liver and heart transplant recipients may, similar to the renal transplant recipients, reflect the presence of both CKD and immune suppression. The implications for this finding are as yet unknown but may potentially include worse cardiovascular prognosis in the setting of immunosuppressive agents.

Data from decades ago suggested a significant presence of mineralization defects in pediatric kidney transplant recipients [22] and while average and median values for parameters of osteoid accumulation and mineralization (i.e. osteoid maturation time and mineralization lag time) were within the upper limits of normal in the current cohort of transplant recipients, a significant proportion of the population had values above the normal range [10], despite low-normal values for bone turnover, suggesting that defects in skeletal mineralization may persist in the current era of immune suppression. It is important to note, however, that while the pre-dialysis CKD patients in the current study were recruited from a cross-section of patients for the research purpose of assessing the spectrum of renal osteodystrophy in the pediatric pre-dialysis CKD population [11], the transplant recipients were selected for bone biopsy based on their history of osteoporotic fractures [10]. Whether these selection criteria contributed to the prevalence of mineralization defects and differences in osteocytic protein expression is unknown, but osteoporotic fractures are common in prevalent solid-organ transplant recipients, in whom peripheral fractures occur in as many as 16% and vertebral fractures in 8% [1]. Thus, the solid-organ recipients selected for the current analysis at the very least represent an important subset of the pediatric transplant population.

Consistent with previous studies [6,23,24], skeletal expression of FGF23 correlated inversely with osteoid accumulation—most particularly, with osteoid thickness. This suggests that higher bone FGF23 in transplant recipients may occur in response to glucocorticoid-mediated changes in bone mineralization, rather than to direct stimulation of osteocytic protein expression. Although high bone turnover was infrequent in the current cohort of patients, precluding testing as to whether FGF23, DMP1, and sclerostin are affected by high bone turnover after solid organ transplantation, no relationship was noted between bone formation rate and expression of any of the osteocytic proteins, consistent with previous data in pre-dialysis CKD and dialysis patients [6,23,24]. Although circulating FGF23 were not assessed in the current study, elevated bone FGF23 expression in recipients of all types of solid organ transplantation should be considered carefully, particularly in light of the accumulating data suggesting that elevated FGF23 levels contribute to cardiovascular morbidity [25], to mortality [26], and to progressive end-organ damage [27].

While DMP1 has been implicated as a down-regulator of FGF23, previous data have suggested that a concomitant upregulation of DMP1 and FGF23 occurs in pediatric CKD, suggesting that DMP1-mediated suppression of FGF23 may be ineffective in this population. In the current study, bone DMP1 expression was increased from normal controls in pre-dialysis CKD patients and in liver and heart allograft recipients, although not in kidney transplant recipients. Whether this finding reflects an interaction between immunosuppressive agents and CKD remains to be determined; however, it is interesting to note that serum phosphorus levels were lower in kidney transplant recipients and phosphorus has been shown to stimulate DMP1 expression [28]. It is also important to note that since the antibody used for immunohistochemical detection of DMP1 in the current study cross reacts with the full-length 98 kDa moiety as well as its fragments [29], increased amounts of total DMP1 in bone may or may not reflect an increase in its activity.

Expression of sclerostin, an inhibitor of Wnt signaling, has been shown to be increased in adults with early CKD [7]. In the current study, the decreased sclerostin expression in the non-transplanted CKD patients as compared to controls may either reflect a difference in osteocyte biology between children and adults or reflect the fact that PTH values, which are known to suppress sclerostin, were already mildly increased in this cohort. However, the increase in cortical sclerostin expression in the transplant recipients—even in kidney transplant recipients with similar circulating PTH values—from the cohort of non-transplanted CKD patients, suggests that immune-suppressive medications act to increase bone sclerostin expression. Interestingly, sclerostin expression did not differ between kidney and other solid-organ allograft recipients, despite differences in PTH values between these two groups. The implications for increased bone sclerostin expression post solid-organ transplantation is currently unknown; however, its role in the inhibition of Wnt signaling, a pathway critical for osteoblast function, could contribute to the 6-fold higher incidence of all fractures and 160-fold higher incidence of vertebral fractures in this population than in the general pediatric population at large [1].

Expression of osteopontin, an inhibitor of skeletal mineralization, did not differ between patients with non-transplant CKD, those status-post kidney transplantation, and those with heart or liver allografts, suggesting that expression of this specific protein may not be altered by CKD or by the presence of immune-suppression. Osteopontin was found in the matrix of mineralized bone, along cement lines, and while its expression was not related to parameters of osteoid accumulation on bone biopsy, it is possible that its expression may be related to the quality and density of mineralized matrix itself, a feature of bone health that is not quantified by classic histomorphometric analysis but which might be revealed through newer imaging techniques, including back-scatter electron microscopy or infra-red techniques and which warrants further investigation.

In conclusion, the current study demonstrates that solid-organ transplantation and immunosuppressive medication post-transplantation are associated with changes in osteocytic protein expression, including increased FGF23 and sclerostin expression, that appear to be distinguishable from the effects of CKD alone on altered osteocyte biology. Although consisting of a relatively small sample size, the current study presents the first human data on the impact of immunosuppressive agents on osteocytic protein expression. The impact that these changes have on skeletal and systemic complications that have been associated with CKD, including progressive renal dysfunction and cardiovascular disease, is unknown but warrants further evaluation.
